# High thromboembolic event rate in patients with locally advanced oesophageal cancer during neoadjuvant therapy. An exploratory analysis of the prospective, randomised intergroup phase III trial SAKK 75/08

**DOI:** 10.1186/s12885-020-6623-z

**Published:** 2020-02-28

**Authors:** Martin Fehr, Hanne Hawle, Stefanie Hayoz, Peter Thuss-Patience, Sabina Schacher, Jorge Riera Knorrenschild, Donat Dürr, Wolfram T. Knoefel, Holger Rumpold, Michael Bitzer, Martin Zweifel, Panagiotis Samaras, Ulrich Mey, Marc Küng, Ralph Winterhalder, Wolfgang Eisterer, Viviane Hess, Marie-Aline Gérard, Arnoud Templeton, Michael Stahl, Thomas Ruhstaller, for the Swiss Group for Clinical Cancer Research (SAKK), the German Esophageal Cancer Study Group, the Austrian Arbeitsgemeinschaft Medikamentöse Tumortherapie (AGMT), the Fédération Francophone de Cancérologie Digestive (FFCD) / Fédération de Recherche en Chirurgie (FRENCH)

**Affiliations:** 1 Department of Medical Oncology and Haematology, Cantonal Hospital St. Gallen, Rorschacherstrasse 95, 9007 St. Gallen, Switzerland; 2 SAKK Coordinating Center, Bern, Switzerland; 3 Charité – Universitätsmedizin Berlin, Berlin, Germany; 4 Kantonsspital Winterthur, Winterthur, Switzerland; 5 Universitätsklinikum Giessen und Marburg, Marburg, Germany; 6 Stadtspital Triemli, Zürich, Switzerland; 7 Universitätsklinikum Düsseldorf, Düsseldorf, Germany; 8 Krankenhaus der barmherzigen Schwestern, Linz, Austria; 9 Landeskrankenhaus Feldkirch, Feldkirch, Austria; 10 Universitätsklinikum Tübingen, Tübingen, Germany; 11 Inselspital Bern, Bern, Switzerland; 12 Universitätsspital Zürich, Zürich, Switzerland; 13 Kantonsspital Graubünden, Chur, Switzerland; 14 Hôpital Fribourgeois, Villars-sur-Glâne, Switzerland; 15 Luzerner Kantonsspital, Luzern, Switzerland; 16 Medizinische Universität Innsbruck, Innsbruck, Austria; 17 Klinikum Klagenfurt am Wörthersee, Klagenfurt, Austria; 18 Universitätsspital Basel, Basel, Switzerland; 19 Claraspital Basel, Basel, Switzerland; 20 Evang. Kliniken Essen-Mitte, Essen, Germany; 21 University of Basel, Basel, Switzerland

**Keywords:** Oesophageal cancer, Adenocarcinoma, Thrombosis, Venous thrombosis, Thromboembolic events, Preoperative therapy, Neoadjuvant therapy, Cisplatin, Chemoradiotherapy

## Abstract

**Background:**

High rates of venous thromboembolic events (VTEs), mainly in advanced disease, are reported for patients with cancer of the upper gastrointestinal tract (stomach, pancreas) and for treatment with cisplatin.

**Methods:**

Exploratory analysis of VTEs reported as adverse events and serious adverse events in a prospective, randomised, multicentre, multimodal phase III trial according to VTEs reported as adverse events and severe adverse events. Patients with resectable oesophageal cancer (T2N1–3, T3-4aNx) were randomized to 2 cycles of chemotherapy with docetaxel 75 mg/m^2^, cisplatin 75 mg/m^2^ followed by chemo-radiotherapy (CRT) and subsequent surgery (control arm) or the same treatment with addition of cetuximab (investigational arm).

**Results:**

VTEs occurred in 26 of 300 patients included in the trial, resulting in an incidence rate (IR) of 8.7% [95% CI 5.7–12.4%]. A total of 29 VTEs were reported:13 (45%) VTEs were grade 2, 13 (45%) grade 3 and three (10%) fatal grade 5 events. 72% (21/29) of all VTEs occurred preoperatively (IR 6.7%): 14% (4/29) during chemotherapy and 59% (17/29) during CRT. In multivariable logistic regression only adenocarcinoma (IR 11.1%, 21/189 patients) compared to squamous cell cancer (IR 4.5%, 5/111 patients) was significantly associated with VTE-risk during treatment, OR 2.9 [95%CI 1.0–8.4], *p* = 0.046. Baseline Khorana risk score was 0 in 73% (19/26), 1–2 in 23% (6/26) and 3 in only 4% (1/26) of patients with VTEs.

**Conclusion:**

A high incidence of VTEs during preoperative therapy of resectable oesophageal cancer is observed in this analysis, especially in patients with adenocarcinoma. The role of prophylactic anticoagulation during neoadjuvant therapy in resectable esophageal cancer should be further evaluated in prospective clinical trials. According to our data, which are in line with other analysis of VTE-risk in patients with oesophageal cancer patients treated with neoadjuvant cisplatin-based chemotherapy and CRT, prophylactic anticoagluation could be considered balanced against individual bleeding risks, especially in patients with adenocarcinoma. In addition to the established risk factors, oesophageal adenocarcinoma treated with neoadjuvant cisplatin-based therapy may be regarded as a high-risk situation for VTEs.

**Trial registration:**

Registered at clinicaltrials.gov*,* NCT01107639, on 21 April 2010,

## Background

Thromboembolic events during cancer therapy may be associated with significant additional morbidity and reduced quality of life in addition to detrimental effects on clinical outcome of individual patients [1]. A variety of inter-related patient-, tumour-, and therapy-related factors contribute to the risk of venous thromboembolic events (VTEs) in cancer patients. Chemotherapy as well as radiotherapy are recognized as independent risk factors for thrombosis and may cause damage to the vascular endothelium and disequilibrium between pro-coagulant and anticoagulant factors [2, 3]. For cisplatin-containing chemotherapy, particularly high incidence rates of VTEs have been reported, mainly from retrospective analyses of heterogeneous patient cohorts and advanced disease: [1, 4–6] In a retrospective single centre analysis an incidence rate of up to 18.1% (169 of 932 patients) has been reported [1]. A systemic review and meta-analysis of randomised controlled trials demonstrated a significantly increased relative risk (RR) of 1.67 (*P* = 0.01) for VTEs in patients with cisplatin-based chemotherapy as compared to those without cisplatin with incidence rates of 1.92% vs. 0.79% [5]. These analyses only included few patients with oesophageal cancer. An exploratory analysis of the REAL-2 study, which included 1002 patients with advanced gastric and gastro-oesophageal junction (GEJ) cancer, demonstrated an incidence of 10,1% overall; the rate was significantly higher in patients treated with chemotherapy-combinations including cisplatin as compared to oxaliplatin: 15.1% vs. 7.6%; *P <* 0.001) [1].

A risk model for VTEs in cancer patients known as the “Khorana risk score” has been established in large derivation and validation cohorts of cancer patients being treated with chemotherapy (*n* = 2701 and *n* = 1365, respectively) [7]. In this model the risk of VTEs is assessed by 5 predictive variables: Site of cancer, high body mass index (BMI), leukocyte (Lc) and thrombocyte count (Plt), haemoglobin level (Hb) or use of red cell growth factor. A high risk is attributed to pancreatic and stomach cancer as primary site of cancer. However, it is unclear whether this high risk also applies for other cancers from the upper gastrointestinal tract, such as locally advanced oesophageal carcinoma as these patients have neither been represented at large in the “Khorana” cohorts nor in similar analyses [1, 7].

Patients with locally advanced and resectable oesophageal cancer are treated with multimodal therapy in curative intention with 5-year overall survival rates of at least 50% and platinum-based chemotherapy is an established part of the treatment [8–10]. Due to the paucity of prospective data on VTEs in resectable oesophageal cancer the aim of this analysis was to describe the rate and pattern as well as potential risk factors of VTEs (such as histologic subtype and the “Khorana risk factors” listed above) in patients with resectable oesophageal cancer with multidisciplinary treatment within an international phase lll trial.

## Methods

### Study design

We conducted an exploratory analysis of VTEs in the international, multicentre intergroup phase III trial SAKK 75/08 according to reported adverse events (AEs) and severe adverse events (SAEs) from start of preoperative treatment until 6 months postoperatively. This additional analysis was planned after initiation of the study but before primary analysis. The study design and clinical efficacy endpoints have been published in detail [8].

### Treatment

In brief, 300 patients with resectable oesophageal cancer (T2N1–3, T3-4aNx) were included and received 2 cycles of induction chemotherapy with docetaxel 75 mg/m^2^ and cisplatin 75 mg/m^2^ (duration of cycle 3 weeks) followed by chemoradiotherapy (CRT) with 45 Gy (1,8 Gy × 25), docetaxel 20 mg/m^2^ and cisplatin 25 mg/m^2^ weekly for 5 weeks and then surgery in the control arm or were randomly assigned to the same treatment with addition of cetuximab preoperatively (400 mg/m^2^ initially, then 250 mg/m^2^ weekly) and postoperatively (250 mg/m^2^ every 2 weeks for 3 months postoperatively) in the investigational arm.

### Objectives

The primary objective of this analysis was to evaluate the incidence rate (IR) of VTEs in patients with resectable oesophageal cancer undergoing multimodality treatment as described above. For this analysis, both study-arms were combined, as the primary endpoint of the study, progression free survival (PFS), was not met. Any VTE - except for superficial thrombophlebitis - which was reported by the investigators as AE and SAE and confirmed by scheduled or unscheduled scans (by any modality as considered appropriate by the investigators according to the individual clinical situation – e.g. sonography, CT-scan, radioisotope scans) was considered as a relevant event. No routine screening for the detection of clinically asymptomatic VTEs was mandated by the study protocol.

Secondary objectives included grades according to “common terminology criteria of adverse events version 4.0” (CTCAE v4.0) and location of VTEs, the incidence of VTEs during different phases of treatment, comparison of VTEs in the control arm vs. investigational arm, VTEs according to histologic subtypes, association with clinical efficacy endpoints and to evaluate whether the “Khorana risk factors “(Hb < 100 g/l or use of erythropoiesis stimulating agents, Lc > 11 G/l, Plt > 350 G/l, BMI > 35 kg/m^2^; excluding site of cancer)^10^ were prevalent in patients with VTEs. The duration of the treatment phases were defined as follows: 6 weeks of induction chemotherapy (total of 2 cycles, duration of each cycle 3 weeks); CRT lasted for 5 weeks and additional 30 days for recovery until the operation (total of 9 weeks and 2 days); postoperative period: A total 6 months after the operation.

### Statistical methods

Continuous data were summarized using median and range. Categorical data were summarized using frequency counts and percentages and compared between subgroups using Fisher’s exact test. Effects of pre-selected covariates (treatment arm, histologic subtypes and Khorana risk factors) on these endpoints were explored using logistic regression. Time-to-event endpoints were summarized by the median and corresponding 95% confidence interval using the Kaplan-Meier method. The number of events was described descriptively by frequency and percentage. All analyses were conducted using SAS 9.4 (SAS Institute Inc.), no adjustment was made for multiple testing and all analyses are considered exploratory.

## Results

### Overall

Demographics and disease characteristics are shown in Table 1.

**Table 1:** Demographics and disease characteristics of the patients included in the trial

Characteristic	Overall (*N* = 300)	
	*N*	(%)
Median age (years), median (range)	61	(36–75)
Sex		
Male	263	(88%)
Female	37	(12%)
Histologic Type		
Adenocarcinoma	189	(63%)
Squamous cell carcinoma	111	(37%)
Localization (main tumour load)		
Upper part (5 cm from thoracic inlet to tracheal bifurcation)	14	(5%)
Lower part (tracheal bifurcation to oesophagogastric junction)	141	(47%)
Oesophagogastric junction	145	(48%)
Siewert Type		
Type I	88	(29%)
Type II	57	(19%)
Clinical T stage		
uT2	44	(15%)
uT3	246	(82%)
uT4a	10	(3%)
Clinical N stage		
N0	31	(10%)
N+	269	(90%)
WHO Performance Status		
0	189	(63%)
1	109	(36%)
Missing	2	(1%)

Of 300 patients included, 29 VTEs were reported in 26 patients corresponding to an IR of 8.7% [95% CI 5.7–12.4%]. Two patients had two VTEs at different time points, whereas one patient had two simultaneous VTEs at separate locations. 72% (21/29) of all VTEs occurred preoperatively: 14% (4/29) during induction chemotherapy, 59% (17/29) during chemo-radiotherapy (CRT). This corresponds to an overall IR of 6.7% (20/300) for the preoperative period of 15 weeks.

**Fig. 1:**
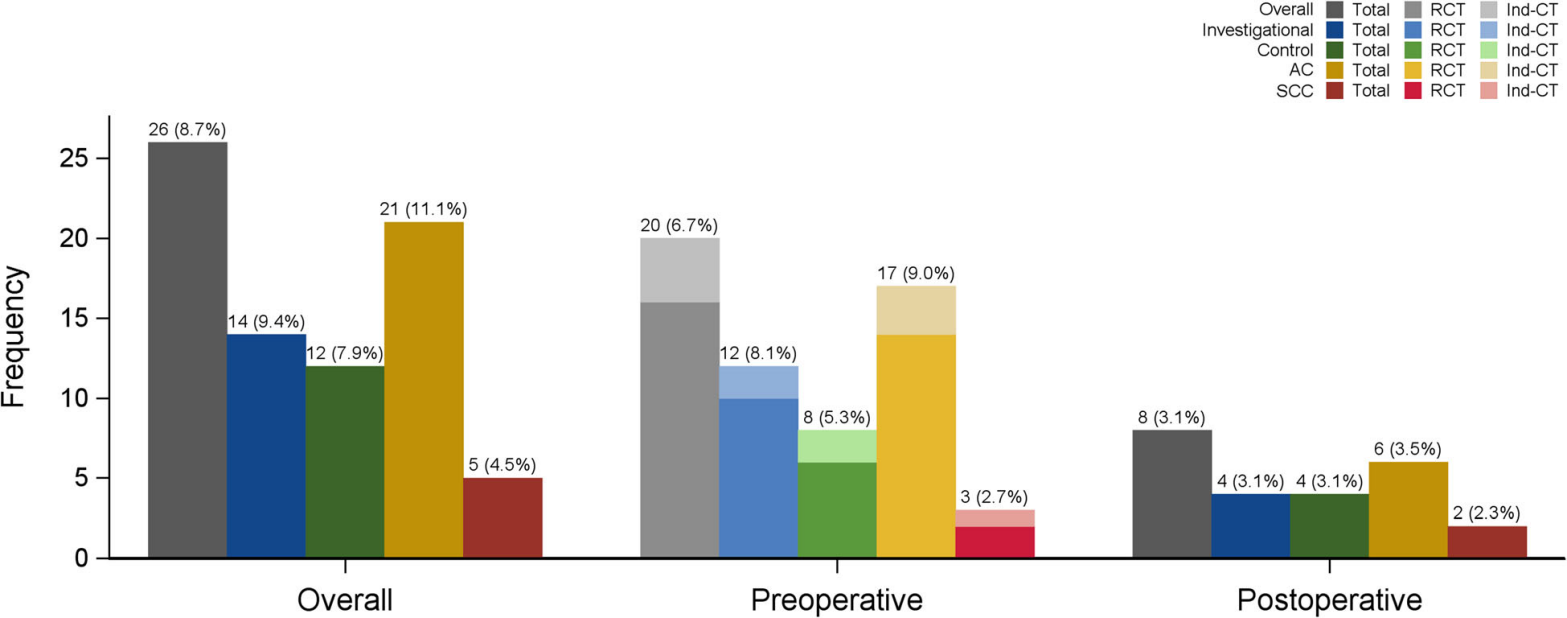
Display of the distribution of VTEs according to treatment arm, histologic subtype and treatment phase

Respectively, 28% (8/29) of all VTEs occurred during the postoperative period of 6 months *(*Fig. 1*,* Table 2*).*

**Table 2:** VTEs according to treatment arm, histologic subtype and treatment phase

	Overall N = 300	Investigational Arm *N* = 149	Control Arm *N* = 151	Adeno-carcinoma *N* = 189	Squamous Cell Carcinoma *N* = 111
Overall (N)	**8.7% (26**^b^**)**	**9.4% (14**^a^**)**	**7.9% (12)**	**11.1% (21**^a^**)**	**4.5% (5)**
Preoperative (N = 300)	**6.7% (20**^b^**)**	**8.0% (12**^a^**)**	**5.3% (8)**	**9% (17**^a^**)**	**2.7% (3)**
Ind-CT	1.3% (4)	1.3% (2)	1.3% (2)	1.6% (3)	0.9% (1)
RCT	5.6% (16^b^)	7.1% (10^a^)	4.1% (6)	7.7% (14^a^)	1.9% (2)
Postoperative (*N* = 259)	**3.1% (8)**	**3.1% (4)**	**3.1% (4)**	**3.5% (6)**	**2.3% (2)**

^a^One of these patients had two simultaneous grade 3 VTEs in the RCT phase

^b^Two of these patients had two separate grade 3 VTEs at different time points

### Location and grades

Ten out of the 21 preoperative VTEs (47%) occurred peripherally (any location that is no pulmonary embolism). In nine (43%) cases pulmonary embolism were observed, which were all grade 2 and 3 and none was fatal, respectively. In two (10%) cases the location was not specified. One patient had two separately located thromboses simultaneously during the preoperative period. Ten (48%) preoperative VTEs were of grade 3, which relates to the need of hospital admission or prolonged hospitalisation. Only one of 29 VTEs was initially asymptomatic and reported as grade 1. Due to the need for medical intervention it was re-classified as grade 2. Two patients with preoperative VTEs had another separate event postoperatively. For further details see Table 3.

**Table 3:** VTEs in different treatment phases and Grades according to CTCAE v4.0

Grade	Overall % (*N* = 29)	Preoperative CT & CRT % (*N* = 21^a^)	Postoperative % (*N* = 8)
G1	0% (0)	0% (0)	0% (0)
G2	45% (13)	52% (11)	25% (2)
G3	45% (13)	48% (10)	38% (3)
G4	0% (0)	0% (0)	0% (0)
G5	10% (3)	0% (0)	38% (3)

Abbreviations: *G* Grade, *CTCAE* Common Toxicity Criteria for Adverse Events

^a^One patient had two separate VTEs in the RCT phase

Of eight VTEs during the postoperative period the location was unspecified in one (12.5%) case, two (25%) were located peripherally and five (62.5%) were pulmonary embolisms; of note, three of these five postoperative pulmonary embolisms were fatal (grade 5).

### Investigational vs. control arm (+/− cetuximab)

During the preoperative period 12 patients (IR 8.0%) in the investigational arm experienced VTEs as compared to eight patients (IR 5.3%) in the control arm (Odds ratio (OR) 1.57 [95% Confidence interval (CI) 0.62–3.95], *p =* 0.3 in univariable analysis).

With inclusion of the postoperative period 14 patients (IR 9.4%) of the investigational arm vs. Twelve patients (IR 7.9%) in the control arm had VTEs (ORs 1.20 [95%CI 0.54–2.69], *p =* 0.7). Thus the difference between the two treatment arms is not significant *(*Tables 2 and 4*,* Fig. 1*).*

**Table 4:** Logistic regression for association of selected baseline variables with occurrence of preoperative VTEs (multivariable model)

	Odds Ratio (95% CI)	*p*-value
Arm (Investigational vs. Control)	1.56 (0.61–4.00)	0.4
Histologic type (AC vs. SCC)	4.42 (1.18–16.53)	0.03
Neutrophils (10^9^/L)	1.14 (0.88–1.47)	0.3
Platelets (10^9^/L)	1.00 (0.99–1.00)	0.2
Haemoglobin (100 g/L)	1.01 (0.98–1.04)	0.7
BMI (kg/m^2^)	0.95 (0.84–1.06)	0.3

### Histologic subtypes

Patients with adenocarcinoma histologic subtype had a higher incidence of preoperative VTEs with 9.0% (17/189 patients) compared to squamous cell carcinoma (SCC) with 2.7% (3/111patients). This difference was statistically significant both in the univariable model (OR 3.56 [95%CI 1.02–12.43], *p =* 0.047) and also in the multivariable model (OR 4.42 [95%CI 1.18–16.53], *p* = 0.03; Tables 2 and 4*,* Fig. 1).

The difference of VTE-risk between histologic subtypes remained statistical significant for the whole study period including the postoperative period in a multivariable model including baseline Hb, thrombocyte count, neutrophils, BMI and treatment arm (Adenocarcinoma 11.1% vs. SCC 4.5%, OR 2.93 [95%CI 1.02–8.44], *p* = 0.046)*.*

### Comparison to the Khorana risk score

The following baseline risk factors (RF) of the Khorana risk score were assessed in patients with VTEs: Hb < 100 g/l or use of erythropoiesis stimulating agents, leucocytes > 11 G/l, Plt > 350 G/l, BMI > 35 kg/m^2^. Oesophageal cancer as site of cancer is not a risk factor in the Khorana risk score. One fifteen of the 20 patients (75%) with preoperative VTEs had no baseline RF and five (25%) had 1–2 RFs, respectively. No patient with preoperative VTE had > 3 risk factors, which would correspond to “high risk” for VTE according to the Khorana risk score. Baseline RFs were > 3 only in one patient (4%; 1/26), who experienced a postoperative VTE.

### Association of VTEs with clinical efficacy endpoints

Fourteen out of 26 patients (54%) with VTEs had a PFS-event (9 progressive disease, 5 deaths) and the median PFS was 2.1 years [95%CI 0.7-not reached] in comparison to the median PFS of the patients without VTEs of 2.5 years [95%CI 1.9–3.7]. Due to the small numbers of events these results should be interpreted with caution.

## Discussion

This exploratory analysis of a large randomized trial in patients with resectable oesophageal cancer receiving multimodal therapy reveals a high IR of VTEs of 6.7% during the perioperative therapy, which is in line with the VTE-rate in “high-risk” patients according to the Khorana risk score [7]. However, none of the patients with preoperative VTEs and only one of the patients with postoperative VTEs would have been identified by the Khorana risk score as “high-risk” with subsequent consideration of prophylactic anticoagulation. Patients with oesophageal adenocarcinoma had pronounced rates of VTE (IR 11% overall and 9% pre-operatively) in comparison to patients with SSC.

The SAKK 75/08 intergroup trial offered an excellent opportunity to analyse the rate of VTEs associated with cisplatin-based chemotherapy and the EGFR-antibody cetuximab. Such an analysis is timely, as EGFR-antibody treatment has recently been attributed to higher risk of VTEs [11]. For example, enrolment of patients into the phase III INSPIRE trial, which evaluated the addition of the anti-EGFR antibody necitumumab to cisplatin-based chemotherapy in patients with metastatic NSCLC, was stopped due to an excess of fatal and nonfatal thromboembolic events and overall number of deaths in the experimental arm [11]. In our study of resectable oesophageal cancer, the incidence of VTEs in the investigational treatment arm with cisplatin, docetaxel and cetuximab was not significantly different compared to the arm without cetuximab (9.4 vs. 7.9%, *p* = 0.7). This is in line with the results of the phase2/3 SCOPE1 trial, which investigated the addition of cetuximab to definitive CRT with cisplatin and capecitabine in patients with non-metastatic, non-resectable oesophageal carcinoma, with rates of 11% (CRT with cetuximab) vs. 9% (CRT only) for grade 3 and 4 thrombosis and embolism [9].

The VTE rate in this cohort of resectable oesophageal cancer, especially in adenocarcinoma (9.0% preoperatively, 11.1% overall), exceeds - in a historical comparison - the rates for other “high-risk” patients according to the Khorana risk score, which are reported at 6.7 and 7.1%, respectively [7]. Also the rates for thromboembolic events in the randomised phase II/III SCOPE1 trial, which investigated the addition of cetuximab to cisplatin and fluoropyrimidine-based definitive CRT in patients with non-resectable oesophageal cancer, were reported to be at a similar high level but without further information on histologic subtypes (9% for CRT only, 11% for CRT plus cetuximab) [9]. Therefore it is reasonable to conclude that patients with locally advanced carcinoma of the oesophagus, especially the adenocarcinoma subtype, undergoing treatment with CRT including cisplatin should be regarded at high risk for VTE independent of the VTE-risk assessment by Khorana risk score.

Our data were prospectively collected from an international controlled clinical trial with uniform reporting and monitoring of AEs and SAEs. In contrast to previous retrospective analysis of thromboembolic events during treatment with cisplatin, we analysed a rather homogenous patient cohort accrued over a limited time period from 2010 to 2013 thus reducing the risk of distortion of results from changes in clinical practice of thromboprophylaxis during the observation period. Over-reporting of clinically asymptomatic events is unlikely as the trial was not primarily designed for the detection of VTEs and did not include routine screening tests for the detection of peripheral thrombosis. According to the trial protocol, only one CT-scan (after CRT) was mandatory during the preoperative treatment period. However, additional imaging tests were allowed according to clinical needs of the individual patients at the discretion of the investigator. It is subject to speculation if more frequent imaging would have either lead to earlier detection of severe VTEs or would have contributed to a higher detection rate of clinically asymptomatic VTEs or would have resulted in an even higher overall IR in this cohort of patients.

No data about the use of concomitant anticoagulation – for either prophylactic or therapeutic indications – were captured. However, prophylactic anticoagulation for patients receiving treatment for resectable oesophageal cancer as outpatients, was neither specifically covered by guidelines nor was recommended by the trial protocol. It is unlikely that unreported administration of thromboprophylaxis in a relevant number of patients may have influenced the results of this analysis. In addition, “normal coagulation” was required as inclusion criteria by the trial protocol. Therefore, patients with pre-existing therapeutic anticoagulation were not included in the trial.

We also analysed whether VTEs were associated with detrimental clinical outcome. The median PFS of patients with VTEs was 2.1 years in comparison to 2.5 years for the whole cohort. This difference is not statistically significant and should not be overinterpreted due to the small number of events.

It remains unclear, whether the conclusions of our analysis can be generalized to other platin-containing regimes and a confirmation of our findings by a prospective study in resectable oesophageal adenocarcinoma would be desirable. Unfortunately, VTE rates were not reported separately in the CROSS trail, which compared surgery alone to carboplatin−/taxane-based CRT followed by surgery in patients with early stage oesophageal cancer [9]. In the SCOPE1 trial, comparable VTE rates of 9–11% were reported for definitive cisplatin-based CRT in a more unfavourable patient population with non-metastatic, non-resectable oesophageal cancer.

In this study most likely a combination of factors – histologic subtype, cisplatin-chemotherapy, radiotherapy and the duration of preoperative treatment – may have contributed to high number of VTEs. The relatively low rate of VTEs in SCC subtype argues against cisplatin as the sole thrombogenic element independent of the histology. Adenocarcinoma of lung, pancreas and other localisations in the gastrointestinal tract GI tract are associated with a high incidence of thromboembolism, which is partly mediated by mucin-related coagulopathy [12, 13]. It is subject to speculation whether similar intrinsic factors are also relevant in oesophageal adenocarcinoma.

The updated guidelines of several societies - such as ASCO, ESMO, ISTH - recommend to consider medical thromboprophylaxis in ambulatory cancer patients at high risk for VTE based on risk score assessment, e.g. Khorana risk score [14–16]. Subgroup analysis of the PROTECHT and SAVE-ONCO study have revealed a clinical meaningful number needed to treat of 15 or a low HR (0.27) to prevent VTEs by anticoagulants vs. placebo for the populations defined as high-risk [17, 18].

Recently, the role of new oral anticoagulants (NOAKs) for the prophylaxis of VTEs in ambulatory cancer patients has been evaluated in two large randomized placebo-controlled trials; in both trials, patients with a Khorana risk score > 2 were included: [19, 20] In the AVERT trial, the majority of patients included had advanced disease and a significant reduction of VTEs from 10.2 to 4.2% (HR 0.41, *p* < 0.001) was demonstrated for medical thromboprophylaxis vs. placebo [19]. A substantial number of patients with stomach and GEJ-cancers was included in the CASSINI trial: During the intervention period a reduction of thromboembolic events (HR 0.4) with a low incidence of bleeding (2% vs. 1%) was demonstrated in favour of medical thromboprophylaxis. However, this risk reduction was not significant for the 180-days trial period (HR 0.66, *p* = 0.10) [20]. In neither of these trials a relevant proportion of patients with early oesophageal cancer seem to have been included. Therefore the role of prophylactic anticoagulation in patients with early oesophageal cancer and preoperative therapy is not clearly evaluated and a prospective evaluation would be clearly desirable in this particular group of patients.

According to our analysis, patients with oesophageal adenocarcinoma are at high risk for VTEs during cisplatin-containing preoperative therapy. This is also supported by data on the VTE-incidence of the SCOPE-trial. Taking into account, that clinical benefits for medical thromboprophylaxis have been demonstrated in other high-risk situations and that a VTE could have negative impacts on the curative treatment in early oesophageal cancer, it is reasonable to conclude that medical thromboprophylaxis carefully balanced against individual bleeding risks could be considered in resectable oesophageal cancer during cisplatin-containing multimodal preoperative treatment, especially adenocarcinoma.

## Conclusions

Ideally, the role of prophylactic anticoagulation in resectable oesophageal cancer, especially adenocarcinoma during cisplatin-containing preoperative therapy, should be further evaluated in prospective clinical trials. In view of the high incidence of VTEs in this exploratory analysis of a prospective multicentre phase III trial and the data of other large prospective trials (e.g. SCOPE1), Oesophageal adenocarcinoma treated with neoadjuvant cisplatin-based chemotherapy and CRT may receive attention as another high-risk situation for VTEs in addition to the established risk factors. Given the potential benefits of prophylactic anticoagulation in other cancer patients at high risk for VTEs, medical thromboprophylaxis carefully balanced against individual bleeding risks could also be considered in resectable oesophageal cancer, especially adenocarcinoma, during cisplatin-containing multimodal preoperative treatment.

## Data Availability

The full protocol and data that support the findings of this study are available from SAKK Coordinating Center in Bern, Switzerland, but restrictions apply to the availability of these data, which were used under license for the current study, and so are not publicly available. Data are however available from the authors upon reasonable request and with permission of SAKK.

## Abbreviations

AEs: Adverse events
AGMT: *Arbeitsgemeinschaft Medikamentöse Tumortherapie* (translated as: Austrian working group for medical tumour-therapy)
ASCO: American Society of Clinical Oncology
BMI: Body mass index
CI: Confidence interval
CRT: Chemoradiation, chemo-radiotherapy
CT: Computed tomography
CTCAE: Common Terminology Criteria for Adverse Events
EC: Ethical committee
ESMO: European Society of Medical Oncology
EU: European Union
FFCD: *Fédération Francophone de Cancérologie Digestive* (translated as: Francophon federation of gastrointestinal oncology)
FRENCH: *Fédération de Recherche en Chirurgie* (translated as: Federation for research in surgery)
GEJ: Gastro-oesophageal junction
GI: Gastro-intestinal
Gy: Gray (units)
Hb: Haemoglobin count
HR: Hazard ratio
IR: Incidence rate
ISTH: International Society of Thrombosis and Hemostasis
Lc: Leucocyte count
NOAKs: New oral anticoagulants
OR: Odds ratio
PE: Pulmonary embolism
PFS: Progression free survival
Plt: Thrombocyte count
RR: Relative risk
SAEs: Severe adverse events
SAKK: Schweizerische Arbeitsgruppe für klinische Krebsforschung (translated as: Swiss working group for clinical cancer research
SAS: Statistical Analysis Systems (computer software)
SCC: Squamous cell cancer
VTE: Venous thromboembolic event
VTEs: Venous thromboembolic events
